# Bronchiolitis, epidemiological changes during the SARS-CoV-2 pandemic

**DOI:** 10.1186/s12879-022-07041-x

**Published:** 2022-01-24

**Authors:** Carmina Guitart, Sara Bobillo-Perez, Carme Alejandre, Georgina Armero, Cristian Launes, Francisco Jose Cambra, Monica Balaguer, Iolanda Jordan, Hospital Network for R. S. V. surveillance in Catalonia, Andrés Antón Pagarolas, Jorgina Vila, Ermengol Coma, Iolanda Jordan, Valentí Pineda, Ester Castellarnau, Mª José Centelles-Serrano, Nuria López, Ingrid Badia Vilaró

**Affiliations:** 1 Pediatric Intensive Care Unit. Hospital Sant Joan de Déu, University of Barcelona, Passeig de Sant Joan de Déu, 2, 08950 Esplugues de Llobregat, Barcelona, Spain; 2 Immunological and Respiratory Disorders in the Pediatric Critical Patient Research Group. Institut de Recerca Sant Joan de Déu, University of Barcelona, Barcelona, Spain; 3 Department of Pediatrics, Hospital Sant Joan de Déu, University of Barcelona, Barcelona, Spain; 4 Pediatric Infectious Diseases Research Group, Institut de Recerca Sant Joan de Déu, CIBERESP. Barcelona, Barcelona, Spain

**Keywords:** Bronchiolitis, SARS-CoV-2, Pandemic, Non-pharmaceutical interventions (NPIs)

## Abstract

**Background:**

Bronchiolitis is the most common viral infection of the lower respiratory tract in infants under 2 years of age. The aim of this study was to analyze and compare the seasonal bronchiolitis peaks before and during the SARS-CoV-2 pandemic.

**Methods:**

Descriptive, prospective, and observational study. Patients with severe bronchiolitis admitted to the Pediatric Intensive Care Unit (PICU) of a referral tertiary hospital between September 2010 and June 2021 were included. Demographic data were collected. Viral laboratory-confirmation was carried out. Each season was analyzed and compared. The daily average temperature was collected.

**Results:**

1116 patients were recruited, 58.2% of them males. The median age was 49 days. Respiratory syncytial virus (RSV) was isolated in 782 cases (70.1%). In April 2021, the first and only case of bronchiolitis caused by SARS-CoV-2 was identified. The pre- and post-pandemic periods were compared. There were statistically significant differences regarding: age, 47 vs. 73 days (p = 0.006), PICU and hospital length of stay (p = 0.024 and p = 0.001, respectively), and etiology (p = 0.031). The peak for bronchiolitis in 2020 was non-existent before week 52. A delayed peak was seen around week 26/2021. The mean temperature during the epidemic peak was 10ºC for the years of the last decade and is 23ºC for the present season.

**Conclusion:**

The COVID-19 pandemic outbreak has led to a clearly observable epidemiological change regarding acute bronchiolitis, which should be studied in detail. The influence of the environmental temperature does not seem to determine the viral circulation.

**Supplementary Information:**

The online version contains supplementary material available at 10.1186/s12879-022-07041-x.

## Background

Acute bronchiolitis is the most common viral infection of the lower respiratory tract in infants [1–7]. Although most children do not require hospitalization, approximately 3% of them are admitted to the hospital, accounting for 18% of total hospital admissions in children under 1 year of age [8–10]. Between 2 and 6% of the hospitalized patients require intensive care for respiratory support, which represents 13% of total PICU admissions, leading to high occupancy during the winter season [9, 11–13].

The seasonal pattern of bronchiolitis can be mainly explained by meteorological factors and the fact that transmission is facilitated by being indoors and in close quarters with others [14, 15]. RSV is by far the main causative viral etiological agent, although many others can cause this condition [13]. These viruses are usually spread by large droplets expelled from airways (which requires close contact) and transmission may also occur through fomites in the immediate environment of the infected person [16].

The COVID-19 outbreak, which started in early December 2019 in Wuhan, was declared a pandemic by the World Health Organization (WHO) on March 11th, 2020 [17]. The causative agent, the virus SARS-CoV-2, is believed to be mainly transmitted through the air, carried on droplets (requiring close contact for transmission) and aerosols. For this reason, social distancing, strict hand hygiene, and wearing masks are non-pharmaceutical interventions (NPIs) that became mandatory worldwide [14, 15]. Moreover, school closures were widespread and many countries around the globe went on complete lockdown [14]. All of these actions may have influenced the transmission of other respiratory viruses [18].

A recent meta-analysis [19] looked at the prevalence of respiratory viruses in children with bronchiolitis in the pre-COVID-19 pandemic era, with RSV being the main cause of bronchiolitis in children, followed by rhinovirus (RV) and bocavirus (BoV). Global surveillance data suggest that the SARS-CoV-2 pandemic is a unique opportunity to obtain insight into the dynamics of various other infectious diseases in childhood, including bronchiolitis [20].

There is scarce data about the impact of SARS-CoV-2 regarding acute severe bronchiolitis, with little information available either on bronchiolitis caused by SARS-CoV-2 or on other bronchiolitis viral etiologies being crowded out.

The aim of this study was to describe and compare the bronchiolitis peak in a PICU, through different seasons, before and during the SARS-CoV-2 pandemic.

## Methods

Epidemiological, clinical, and microbiological data were prospectively collected in a database created in 2010. Infants with severe bronchiolitis admitted to the PICU of a tertiary referral hospital (Sant Joan de Déu Hospital, Barcelona) from September 2010 to June 2021 were included. This study was approved by the institutional Clinical Research Ethics Committee and was performed in compliance with the Declaration of Helsinki.

The following variables were included: age, sex, medical history, and previous comorbidities. Risk scores at admission and assessed severity of the bronchiolitis determined using the Bronchiolitis Score of Sant Joan de Déu (BROSJOD) [21] and the Pediatric Risk of Mortality Score III scale (PRISM III) [22]. The support required in the PICU, meaning the need for respiratory support, such as non-invasive ventilation (NIV) (excluding high flow oxygen therapy), and conventional mechanical ventilation (CMV), and for inotropic support. The length of stay (LOS) in the PICU and overall in the hospital. Mortality was recorded as any death occurring during the 28 days following PICU admission.

Determining the causal viral agent for the bronchiolitis was carried out using nasopharyngeal aspirate or a tracheal aspirate/bronchoalveolar lavage (in intubated patients) with a multiplex polymerase chain reaction (PCR) assay (FilmArray®). The SARS-CoV-2 determination was carried out using nasopharyngeal swap/aspirate by real-time reverse transcription-PCR (RT-PCR) in each patient with respiratory symptomatology, since the beginning of the SARS-CoV-2 pandemic.

The different seasons were analyzed and compared. Each season was determined to span from the beginning of September to the end of June of each year. The peak of each season was defined on week 52, in accordance with other published literature [23]. The daily average temperature was collected from the El Prat airport meteorological station, near Hospital Sant Joan de Déu and its demographic reference area. The mean monthly temperature of each season was analyzed. This data was obtained from the Surveillance Network and Air Pollution Forecast from the Government of Catalonia (*La Generalitat*).

Our research group, together with other regional Catalan hospitals, contributes to collecting data from patients with bronchiolitis admitted to each hospital, for the epidemiological surveillance network of Catalonia. In this way, data from all pediatric patients with acute severe bronchiolitis attributed to RSV who were admitted to the PICUs of Catalonia and Europe can be compared.

The aim of this study was to describe and compare the bronchiolitis peak in a PICU, through different seasons, before and during the SARS-CoV-2 pandemic. Secondary objectives were to analyze the demographic characteristics, outcomes and temperature relationships.

The statistical analysis was executed using SPSS® 25.0 software. Categorical variables were expressed as absolute and relative rates, while continuous variables were expressed as median and interquartile range (IQR). The comparison of categorical variables was performed using the χ^2^-test and continuous variables were compared using the Mann–Whitney U-test. The significance level was set at 0.05.

## Results

A total of 1116 patients with severe bronchiolitis were included, 58.2% (650 patients) of them being males. The median age at admission was 49 days (IQR 26–100). No previous comorbidities were described in 777 patients (69.6%). The median BROSJOD severity scale score was 9 points (IQR 7–11) and the median score on the PRISM III scale was 0 points (IQR 0–3). Most patients required intensive respiratory assistance: 1054 patients needed NIV (94.4%), while fewer patients required CMV (381 cases, 34.1%). Of the total, 143 patients needed inotropic support (12.8%). The median PICU LOS was 6 days (IQR 4–11) and the median hospital LOS was 12 days (IQR 8–19). Mortality occurred in 13 (1.2%) patients.

**Fig. 1:**
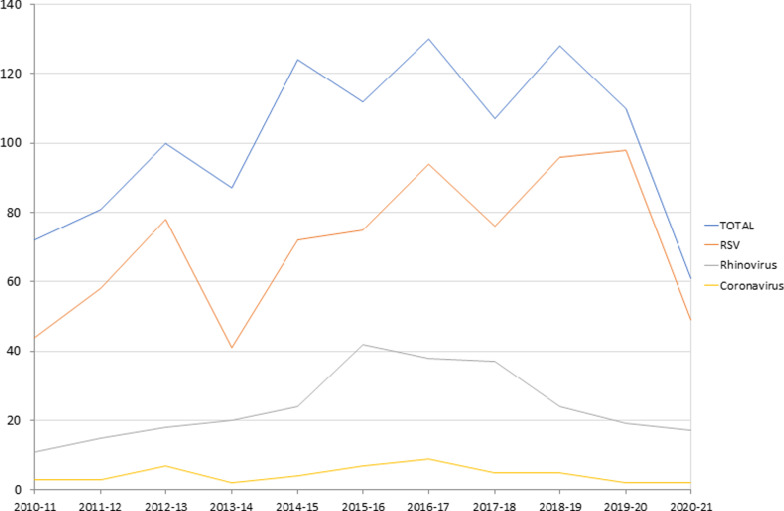
Analysis of viral incidence for each year

Regarding the viral etiology, 782 cases (70.1%) were due to RSV, followed by RV in 265 (23.7%) patients. There were 49 cases (4.4%) caused by seasonal coronaviruses other than SARS-CoV-2. The analysis of the viral incidence for each year is shown in Fig. 1.

**Fig. 2:**
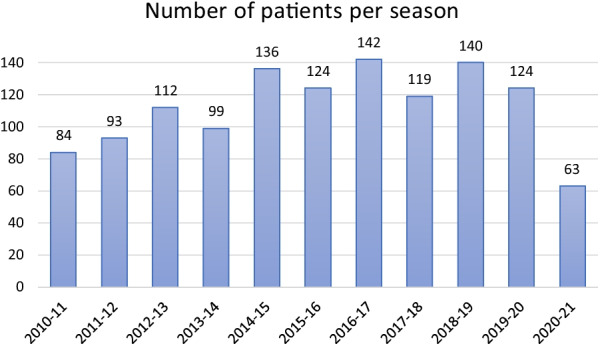
The total yearly number of bronchiolitis patients admitted to the PICU for each season, from 2010 to 2021

**Fig. 3:**
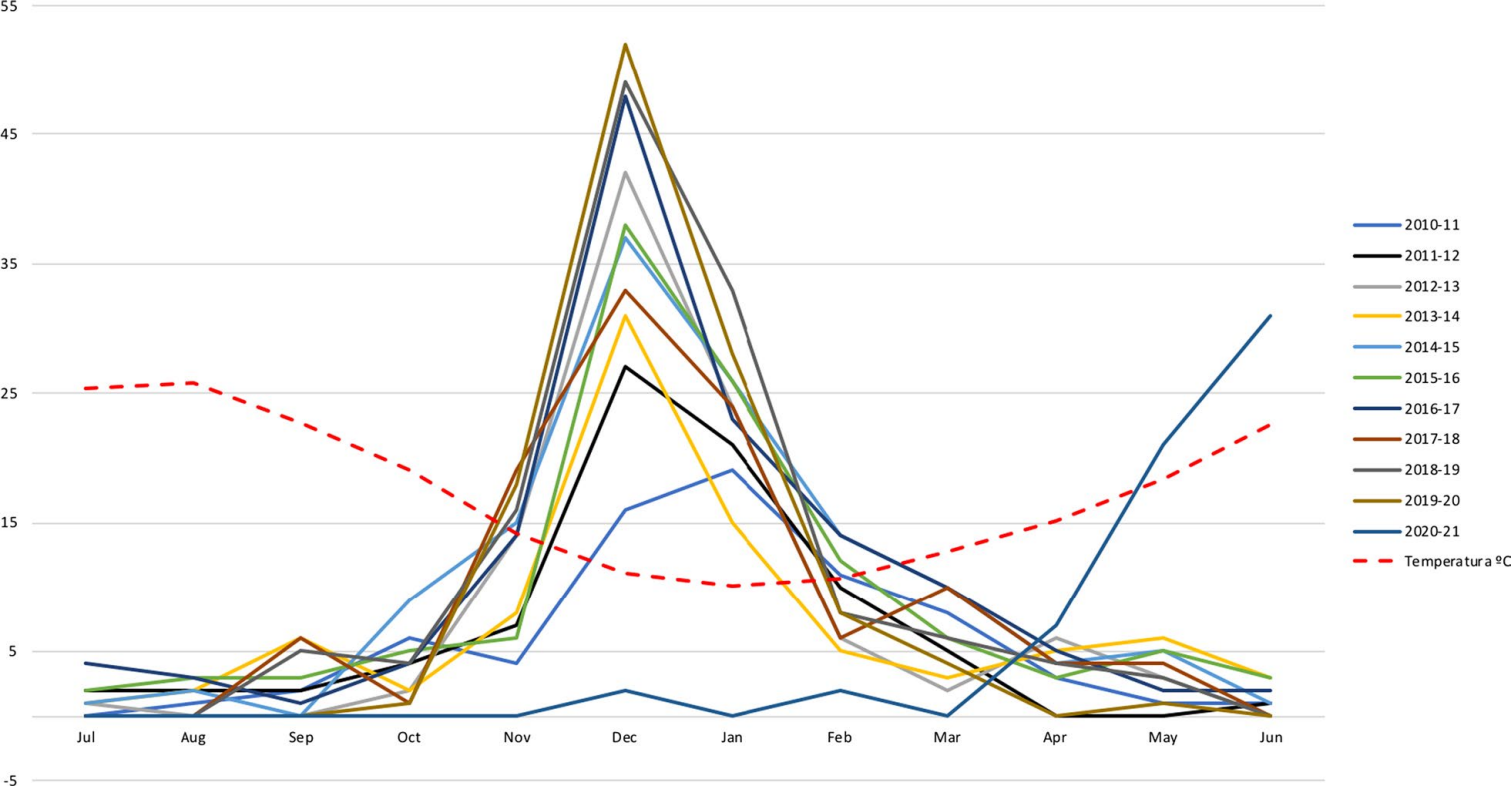
The incidence curve of each season showing the peak, from 2010 to 2021, with the mean temperature for the month

**Fig. 4:**
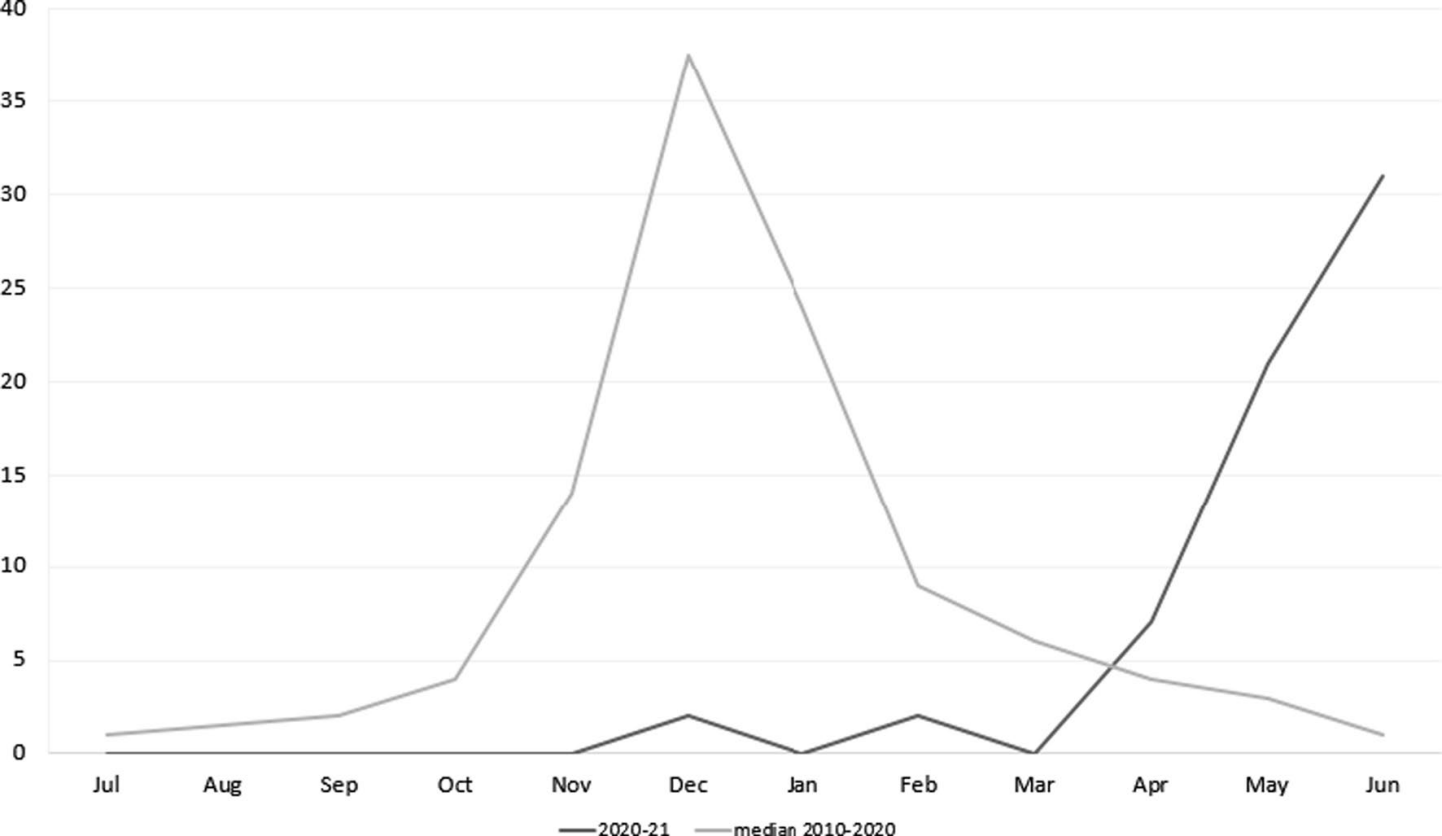
Comparison between the mean of the incidence curve showing the peak from 2010 to 2020 and the incidence curve showing the peak for 2021

The total yearly number of bronchiolitis patients admitted to the PICU each season is shown in Fig. 2. The incidence curve for each season is shown in Fig. 3. Figure 4 illustrates the comparison between the median of the incidence curve from 2010 to 2020 and the incidence curve for 2021.

Lockdown in Catalonia was established from 14th of March to 2nd of May of 2020; followed by periods of opening/closing of restaurants, bars and social activities, depending on the SARS-CoV-2 rates. The use of mask has been mandatory until the present.

The pre- and post-pandemic periods were compared. There were statistically significant differences regarding the following variables: age, 47 days (IQR 26–97) vs. 73 (IQR 33–163), p = 0.006; PICU LOS, 6 days (IQR 4–11) vs*.* 5 (IQR 3–9), p = 0.024; hospital LOS, 12 days (IQR 8–19) vs. 10 days (IQR 7–13), p = 0.001; and etiology, p = 0.031. All the results compared the pre- and post-pandemic period, respectively. The data stemming from additional demographical, clinical and microbiological data are shown in Table 1.

**Table 1:** Demographic, clinical and microbiological data

	TOTAL N = 1116	Pre-pandemic* N = 1053	Post-pandemic** N = 63	p-value
Gender (male), n (%)	650 (58.2)	618 (58.7)	32 (50.8)	0.217
Age (days), median (IQR)	49 (26–100)	47 (26–97)	73 (33–163)	0.006
Comorbidities, n (%)				0.198
None	777 (69.6)	728 (69.1)	49 (77.8)	
Prematurity	241 (21.6)	233 (22.1)	8 (12.7)	
Cardiopathy	73 (6.5)	69 (6.5)	4 (6.3)	
Pneumopathy	33 (2.9)	29 (2.7)	4 (6.3)	
Neuropathy	29 (2.6)	25 (2.4)	4 (6.3)	
Other	55 (4.9)	54 (5.1)	1 (1.6)	
Severity upon admission,				
BROSJOD, median (IQR)	9 (7–11)	9 (7–11)	9 (8–11)	0.262
PRISM III, median (IQR)	0 (0–3)	0 (0–3)	0 (0–3)	0.543
Respiratory support				0.777
NIV, n (%)	1054 (94.4)	995 (94.5)	59 (93.7)	
Days, median (IQR)	3.3 (2–5.1)	3.5 (2–5.6)	3.2 (2–4.8)	0.337
CMV, n (%)	81 (34.1)	363 (34.5)	18 (28.6)	
Days, median (IQR)	7.9 (5.5–10.6)	8 (5.6–11.3)	7.7 (5.5–9.9)	
Inotropic support, n (%)	143 (12.8)	138 (13.1)	5 (7.9)	0.233
Length of stay				
PICU (days), median (IQR)	6 (4–11)	6 (4–11)	5 (3–9)	0.024
Hospitalization (days), median (IQR)	12 (8–19)	12 (8–19)	10 (7–13)	0.001
Death, n (%)	13 (1.2)	13 (1.2)	0 (0)	0.375
Viral etiology, n (%)				0.031
RSV	782 (70.1)	733 (69.6)	49 (77.8)	
RV	265 (23.7)	248 (23.6)	17 (26.9)	
Parainfluenza	59 (5.3)	51 (4.8)	8 (12.7	
Coronavirus	49 (4.4)	47 (4.5)	2 (3.2)	
Adenovirus	34 (3)	31 (2.9)	3 (4.8)	
Bocavirus	16 (1.4)	15 (1.4)	1 (1.6)	
SARS-CoV-2	1 (0.1)	0 (0)	1 (1.6)	

^*^Period since 2010/11 to 2019/20 seasons. **Period during 2020/21 season. n (%): number (percentage). BROSJOD: Bronchiolitis Score of Sant Joan de Déu. PRISM III: Pediatric Risk Score of Mortality III scale. PICU: pediatric intensive care unit. NIV: non-invasive ventilation. CMV: conventional mechanical ventilation

When dividing the total annual number of bronchiolitis cases into those occurring before the peak and those occurring after the peak, which is established at week 52, a sharp decrease in cases was seen for this last season. These results are shown in Table 2. The 2020 bronchiolitis peak is thus non-existent before week 52.

**Table 2:** Number of hospitalized bronchiolitis cases before the peak* and after the peak** flow in PICU

SEASONS	Total (n)	WEEK 52		WEEK 5	
		Before W52	After W52	Before W5	After W5
2010–2011	72	27	45	50	23
2011–2012	81	39	42	62	19
2012–2013	100	53	47	82	18
2013–2014	87	46	41	62	25
2014–2015	124	61	63	89	35
2015–2016	112	50	62	81	31
2016–2017	130	67	63	97	33
2017–2018	107	58	49	84	23
2018–2019	128	73	55	109	19
2019–2020	112	69	43	101	11
2020–2021	63	2	61	3	60

Number of hospitalized bronchiolitis cases before and after the week 5 in PICU

^*^Before the end of the peak = from the beginning of September until week 52 onwards, i.e. the latest end of the peak. **After the end of the peak = from week 53 until the end of June

(n): number. W52: week 52. W5: week 5

The average monthly temperature of each season was analyzed. The mean temperature during the peak for the last decade was 10ºC. The mean temperature during the peak for this last season was 23 ºC. Details for the mean temperatures are included in Fig. 3.

Data on all pediatric patients with acute severe RSV-related bronchiolitis admitted to the PICUs of Catalonia and Europe were consulted and compared (Additional file 1). The incidence and the peak of RSV-attributed bronchiolitis, both for the previous decade and for the last season, are in line with our results. Details on this are shown in Additional file 2.

## Discussion

This study has conclusively shown the very low burden of severe bronchiolitis during the SARS-CoV-2 pandemic and the delayed peak compared with other years in the last decade. Bronchiolitis has always been thought to be related with low temperatures and other meteorological conditions. Also, that throw children begin both RSV and influenza virus epidemics. However, this season breaks all the previously established rules. Due to the distribution of cases seen during this last season, the need has emerged for pediatricians to reconsider those previous theories.

A systematic review and meta-analysis of the prevalence of common respiratory viruses in children younger than 2 years with bronchiolitis in the pre-COVID pandemic era has been published recently [19]. It included 50 articles published between October 1999 and December 2017. RSV was largely the most commonly detected virus (59.2%), both as the only virus involved and in co-infections. In our study, upon analyzing the last ten years before SARS-CoV-2, the same results were found, with RSV being the most prevalent virus in patients with bronchiolitis (70.1%) [19].

As for bronchiolitis due to SARS-CoV-2, coronaviruses other than SARS-CoV-2 are also sometimes detected in respiratory samples, often presenting as co-infections. However, in contrast to what was reported by Milani PG et al [24], we did not find any cases of bronchiolitis due to SARS-CoV-2 during the 2019–2020 season. Instead, we identified the first case of SARS-CoV-2-related bronchiolitis in the 15th week of the current season (2020–2021). No other viruses were isolated for that patient. Recently, a multicenter study of children hospitalized with SARS-CoV-2 [25], concludes that SARS-CoV-2 infection does not seem to be a main trigger of severe bronchiolitis, and that children with this condition should be managed following the clinical practice guidelines.

In general, it has been described that children are less likely to become severely ill from SARS-CoV-2, especially when it comes to respiratory involvement. In Switzerland, one case of life-threatening bronchiolitis was diagnosed [26]. Two other cases were reported in France [27], both presenting with poorly tolerated high fever and neurological symptoms, and they developed acute bronchiolitis within 2 to 8 days. None of them required mechanical respiratory support other than supplemental oxygen.

Few cases of SARS-CoV-2 bronchiolitis or children with severe respiratory symptoms have been described. However, a recent cross-sectional seroprevalence concluded that children appear to have similar probability as adults to become infected by SARS-CoV-2 in quarantined family households, although they remain largely asymptomatic once infected [28].

Last year, some authors analyzed the prevalence of hospitalizations for respiratory diseases in childhood in recent years, with the aim of assessing the impact of social isolation due to COVID-19 on the seasonal behavior of respiratory tract diseases [29–32]. As seen in our results, Nascimento MS et al. [29] observed that the measures adopted during the SARS-CoV-2 pandemic dramatically interfered with the seasonality of childhood respiratory diseases, reflected in the drastic 45% reduction in the number of hospitalizations and reduction in the length of hospital stay, both with statistically significant differences. What is even more remarkable is the finding that there was a reduction in bronchiolitis among the admitted patients from 7.3 to 0% during the social isolation period. Brisca et al. [33] also reported an overall drop in the total number of pediatric consultations in the emergency department of a tertiary Italian hospital, emphasizing a significant decrease in bronchiolitis cases, from 57 during 2019, to 21 during the 2020 lockdown (p < 0.01). Other recently published studies also confirm those findings and strongly suggest that the state of emergency, social distancing and other lockdown strategies are effective at slowing down the spread of respiratory diseases and decreasing the need for hospitalization among children [34–39]. All these results may influence future decision-making aiming to modulate the epidemiology of pediatric diseases.

In Belgium, some authors reported that stay-at-home orders, social distancing, face masks, and other NPIs not only impacted COVID-19, but also the dynamics of various other infectious diseases, having a high impact on seasonal bronchiolitis admissions in pediatric departments worldwide [23]. As is the case with our results, they found an over 99% reduction in the number of recorded RSV cases. However, even though the winter bronchiolitis peak was virtually nonexistent, they feared a delayed spring/summer bronchiolitis peak when most NPIs were relaxed and pre-pandemic life resumed. This became a reality, as shown in the results of our study, in the results from the Catalan region (Additional file 1), and in some other European countries [40] (Additional file 2).

On the other hand, it is interesting to point out, that bronchiolitis decline was mainly due to RSV. Instead, RV had been circulating with relative ease during the lockdown. Although a large decrease in the incidence of bronchiolitis due to RV has been observed, this decrease has been smaller than for RSV despite all isolation measures.

SARS-CoV-2 began to spread throughout the world at a time when the RSV and influenza season was expected to begin in the southern hemisphere. Afterwards, during their summer season, some countries in that hemisphere saw a rising incidence of bronchiolitis, mostly due to RSV, coinciding with the relaxation of social distancing measures. Nascimento et al [29] described an unexpected reduction in the number of hospitalizations in the pediatric population during the usual bronchiolitis season, which they attributed to the social isolation measures adopted. They worried about how 2021 would be. As seen in our results, bronchiolitis reappeared even though the meteorological conditions completely differ from the previous seasons. During this past season we saw a delayed peak of bronchiolitis, which appeared to be related with the withdrawal of social distancing measures.

The authors suggest that hypotheses to explain this late bronchiolitis peak could involve the reduction of isolation measures and the viral interference due to the global spread of a new virus during the same epidemiological period. It is expected that bronchiolitis will continue to be the annual epidemic disease among children under 2 years of age, filling up hospital beds and entailing a global health challenge. Further studies are needed to resolve these questions.

The main limitation of this study is its single-center design. However, a large sample of subjects was included, which permitted the comparison of homogeneous populations. A multi-center study could provide a wider view of the evolution of bronchiolitis during the last year and help explain how a classic childhood epidemic disease virtually disappeared in the context of the SARS-CoV2 outbreak and reappeared afterwards in completely different environmental conditions. The results obtained pointed towards the relevance of the isolation measures in decreasing respiratory viral infections among the pediatric population.

## Conclusion

To sum up, a clear epidemiological change has been observed due to the outbreak of the COVID-19 pandemic. These changes should be studied in detail. The influence of the environmental temperature does not seem to have induced the viral distribution seen this last year, related to severe bronchiolitis, and thus other factors beyond temperature should be investigated. New studies should be considered to delve deeper into this analysis.

## Supplementary Information

**Additional file 1.** Data of the acute severe RSV-related bronchiolitis admitted to the PICUs in Catalonia and Europe.

**Additional file 2.** The incidence and the peak of RSV-attributed bronchiolitis: Temporal comparison.

## Data Availability

Requests for data should be made to the corresponding author. Each request requires a research proposal including a clear research question and proposed analysis plan. Requests will be considered on an individual basis and are subject to review and approval by management committee human research ethics committees.

## Abbreviations

BoV: Bocavirus
BROSJOD: Bronchiolitis Score of Sant Joan de Déu
CMV: Conventional mechanical ventilationÿ
LOS: Length of stay
NIV: Non-invasive ventilation
NPIs: Non-pharmaceutical interventions
PICU: Pediatric intensive care unit
PRISM III: Pediatric Risk of Mortality Score III scale
RSV: Respiratory syncytial virus
RV: Rhinovirus
