# Supplementary material for: Bronchiolitis, epidemiological changes during the SARS-CoV-2 pandemic
Source: BMC Infect Dis. 2022 Jan 24;22:84. doi: 10.1186/s12879-022-07041-x (PMC8785150; doi:10.1186/s12879-022-07041-x)
Supplement: Supplementary file 1 — Additional file 1. Data of the acute severe RSV-related bronchiolitis admitted to the PICUs in Catalonia and Europe. [file 12879_2022_7041_MOESM1_ESM.docx]

1.
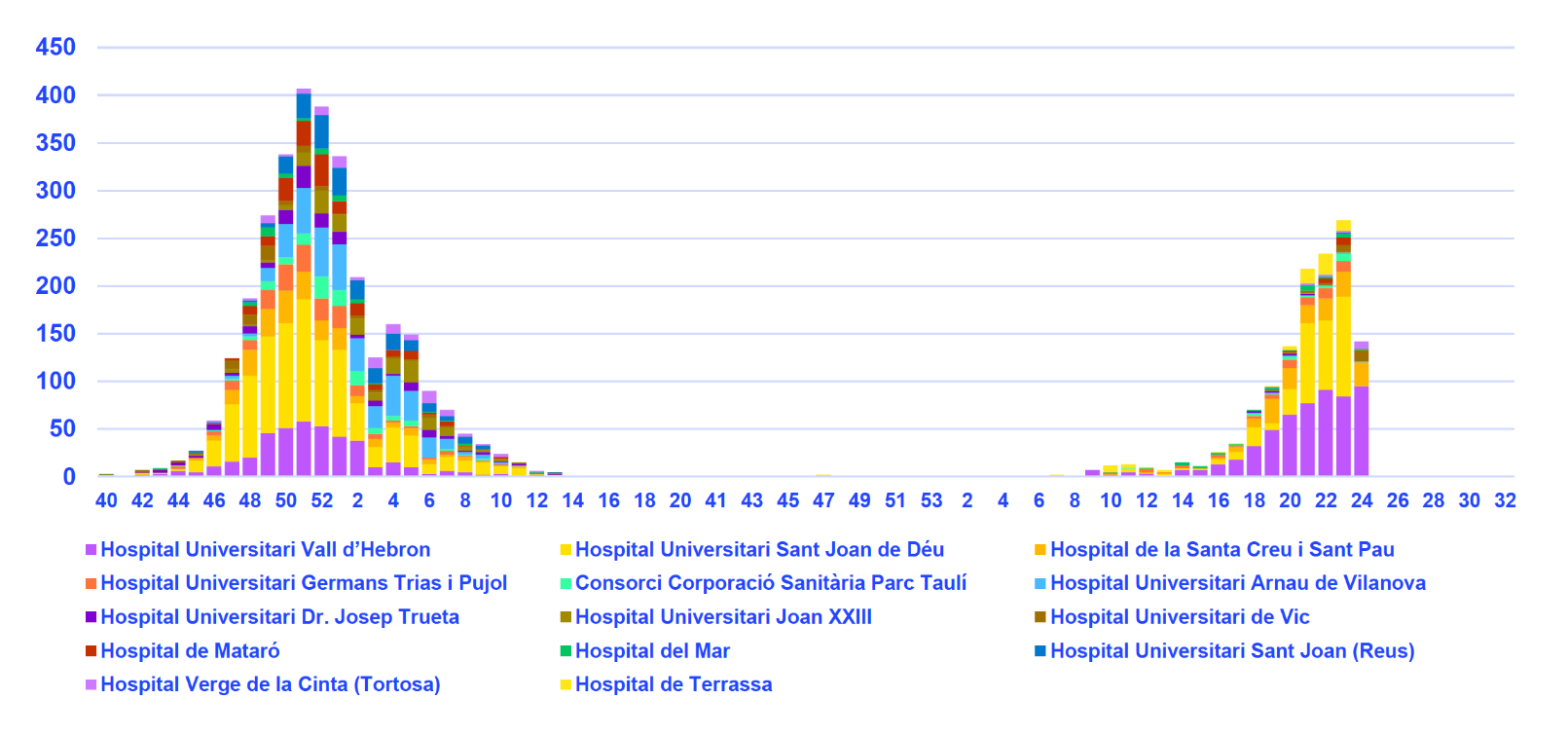
Hospital Surveillance Network of RSV in Catalonia (update date 22/06/2021).
2.
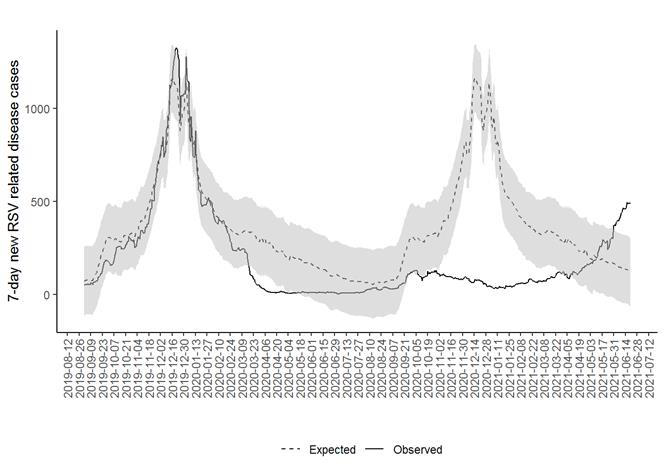

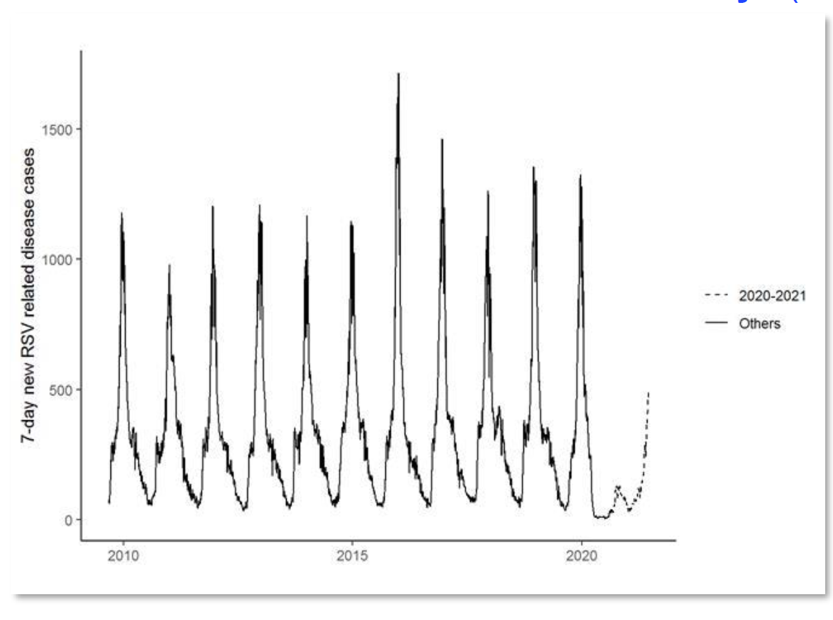
Monitorization of patients younger than 5 years old with bronchiolitis, in the Primary Care (update date 22/06/2021).
