# Supplementary material for: Bronchiolitis, epidemiological changes during the SARS-CoV-2 pandemic
Source: BMC Infect Dis. 2022 Jan 24;22:84. doi: 10.1186/s12879-022-07041-x (PMC8785150; doi:10.1186/s12879-022-07041-x)
Supplement: Supplementary file 2 — Additional file 2. The incidence and the peak of RSV-attributed bronchiolitis: Temporal comparison. [file 12879_2022_7041_MOESM2_ESM.docx]

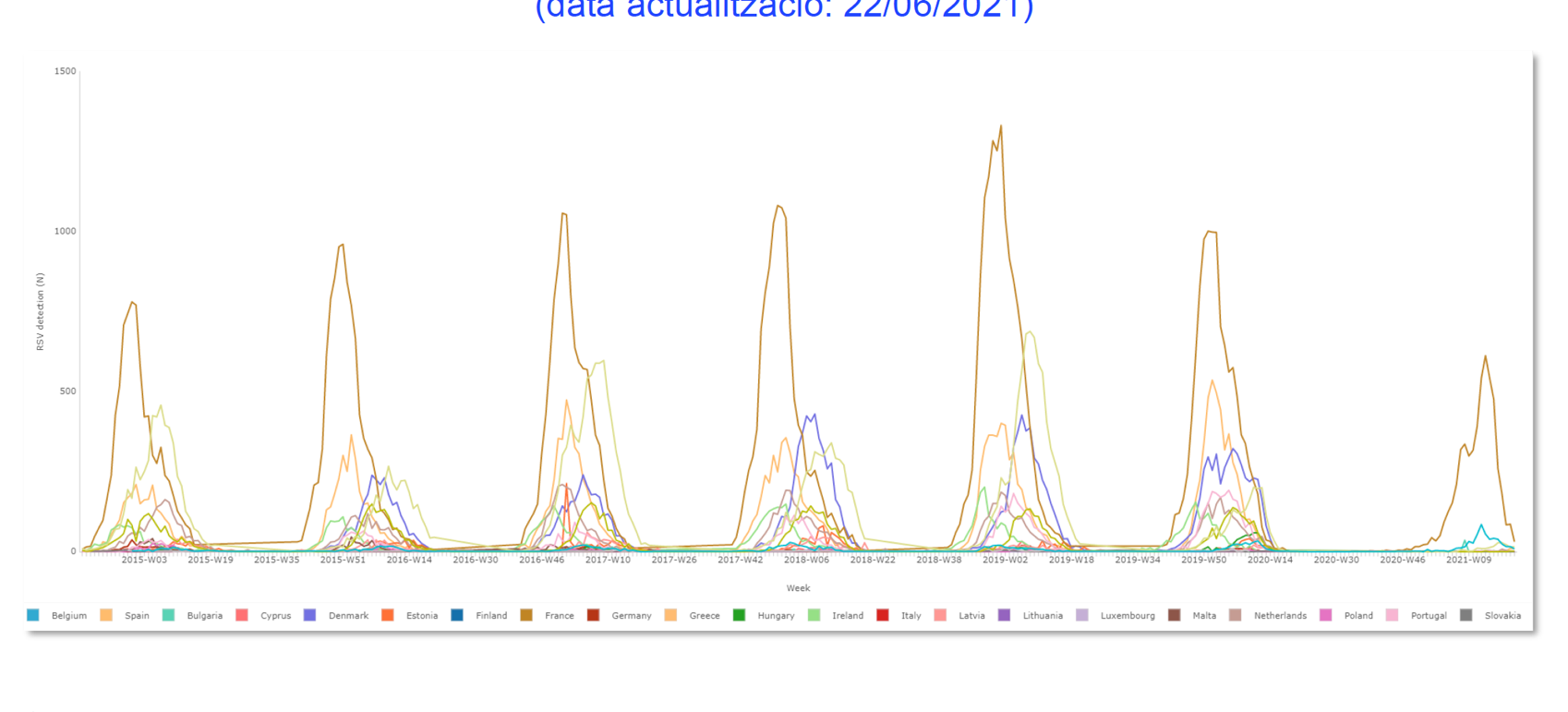
3. Europe situation of the non-sentinel cases of detected RSV (update date 22/06/2021). *http://atlas.ecdc.europa.eu/public.index.aspx*
